# Treatment of Mucous Cyst of the Distal Interphalangeal Joint With Osteophyte Excision and Joint Debridement

**DOI:** 10.3389/fsurg.2021.767098

**Published:** 2022-01-25

**Authors:** Zhiyi Fan, Li Chang, Xing Su, Binbin Yang, Zhe Zhu

**Affiliations:** ^1^Department of Hand Surgery, The Second Hospital of the Jilin University, Changchun, Jilin, China; ^2^Department of Spine Surgery, Affiliated Hospital of Chengde Medical University, Chengde, China; ^3^Department of Pathology, The Second Hospital of Jilin University, Jilin, China; ^4^Department of Thrombosis and Hemostatic, State Key Laboratory of Experimental Hematology, Tianjin Clinical Research Center of Blood Diseases, Institute of Hematology & Blood Diseases Hospital, Chinese Academy of Medical Sciences & Peking Union Medical College, Tianjin, China; ^5^Office of Academic Research, Wenzhou Medical University, Wenzhou, China

**Keywords:** mucous cyst, distal interphalangeal joint, osteophyte excision, joint debridement, post-operative

## Abstract

**Background:**

Mucous cyst of the distal interphalangeal joint (DMC) or interphalangeal connection of the thumb is common in middle-aged and elderly people, and it often occurs in the fingers of people with osteoarthritis (OA). Although there are many conservative treatments, DMC is usually treated by surgery. The common complications of surgical treatment are recurrence of DMC and skin necrosis. This article introduces the method and clinical effect of osteophyte excision and joint debridement in the treatment of DMC of the distal interphalangeal (DIP) joint.

**Methods:**

In total, 19 cases of affected fingers made an 'S' incision in the DIP joint under local anesthesia to remove the osteophyte of the DIP joint, clean the dorsal joint capsule, wash the joint, and retain only the bilateral collateral ligament and extensor tendon device. It is suspected that the injured finger of the extensor tendon should be protected by external fixation.

**Results:**

Out of 15 patients, 1 patient presented with partial skin necrosis that healed after dressing changes while the other patients recovered well. The visual analog scale (VAS) scores of all affected fingers after surgery were lower than those before the surgery (VAS score: 4.93 ± 0.88 vs. 4.07 ± 1.03, *p* < 0.05). The range of motion (ROM) of the affected finger decreased in one patient, and the post-operative activity of the other fingers increased in varying degrees (ROM: 67.60 ± 5.40 vs. 71.27 ± 7.06, *p* > 0.05).

**Conclusions:**

Using osteophyte excision and joint debridement to treat DMC can avoid skin necrosis caused by cyst removal and can avoid the recurrence of DMC to the greatest extent, so it is a safe and effective way of treatment.

## Introduction

The mucous cyst of the distal interphalangeal joint (DMC), known as a joint tendon sheath cyst, is one of the common hand tumors seen in clinic (1). DMC is more common in the middle-aged and elderly population, especially in women, and is related to joint degeneration and osteoarthritis (OA). Degenerative changes in the fibrous bursa of the joint or synovial tissue, especially the increase of synovial fluid after OA and the communication between the cyst and the adjacent joint space have been confirmed to be the important causes of DMC (2–4).

A mucous cyst of the distal interphalangeal joint usually presents as a round or oval mass protruding from the distal interphalangeal (DIP) joint or under the nail. The content is jelly-like and transparent to yellow in color, consisting of hyaluronic acid, globulin, glucosamine, and albumin (3). Although the patient is usually asymptomatic, it may lead to aesthetic problems, such as nail deformities; at the same time, the pain caused by interphalangeal arthritis urges the patient to come to the hospital for treatment (3). DMC is considered a pseudocyst because it does not contain a true epithelial cyst wall and cyst wall. It is formed by the protruding of the articular capsule, while the lining of the DMC is actually formed by fibroblasts surrounded by compressed connective tissue (5), which contains a myxoma matrix with scattered fibroblasts (7). The cyst of the DIP joint of the finger can rupture and cause the gelatinous material to enter the surrounding tissue from the lesion or flow out of the capsule wall. In this case, the cyst will heal and fill within a few weeks, resulting in a recurrence of DMC, so it is difficult to achieve full recovery (3–8).

Mucous cyst of the distal interphalangeal joint usually requires treatment, conservative treatments, such as puncture, sclerotherapy, steroid injections, hyaluronidase injections, cryosurgery, and carbon dioxide vaporization, and various surgical treatments (6, 9–12). So far, there is still no agreement on the best treatment for DMC. According to the systematic review published by Jabbour et al., the effect of surgical treatment is better than that of conservative treatment (5).

There are many surgical treatments, such as removal of cysts, followed by skin grafts or skin flap coverage, or removal of osteophytes and pedicles instead of cysts (8, 13–15). If the incision cannot be stitched directly after the removal because the cyst is too large, skin grafting or flap repair is needed to repair the wound; therefore, the recurrence rate is high (13–15). On the basis of removing osteophytes, we thoroughly remove the degenerative tissues, such as the dorsal articular capsule of the finger, so as to minimize the post-operative recurrence rate.

## Methods

### Patients

Our study included 15 patients who came to our hospital between 2017 and 2020. Patients who met the following criteria were included in the study: (1) patients diagnosed with DMC (1); (2) patients with a strong demand for surgical treatment; and (3) patients with a follow-up >12 months. People who met the following criteria were excluded: (1) those with no range of motion of the affected interphalangeal joint and (2) those with a follow-up period of fewer than 12 months. Of the 15 patients, there were 6 men and 9 women. Mucous cysts occurred in 19 sites of the fingers of these 15 patients. There were seven cases of the thumb, two cases of the index finger, eight cases of the middle finger, and two cases of the ring finger. Except for the thumb mass located on the dorsal side of the interphalangeal joint, the remaining mucoceles were all located on the dorsal side of the DIP joint. X-ray showed different degrees of osteoarthritis.

### Surgery Procedure

**Figure 1:**
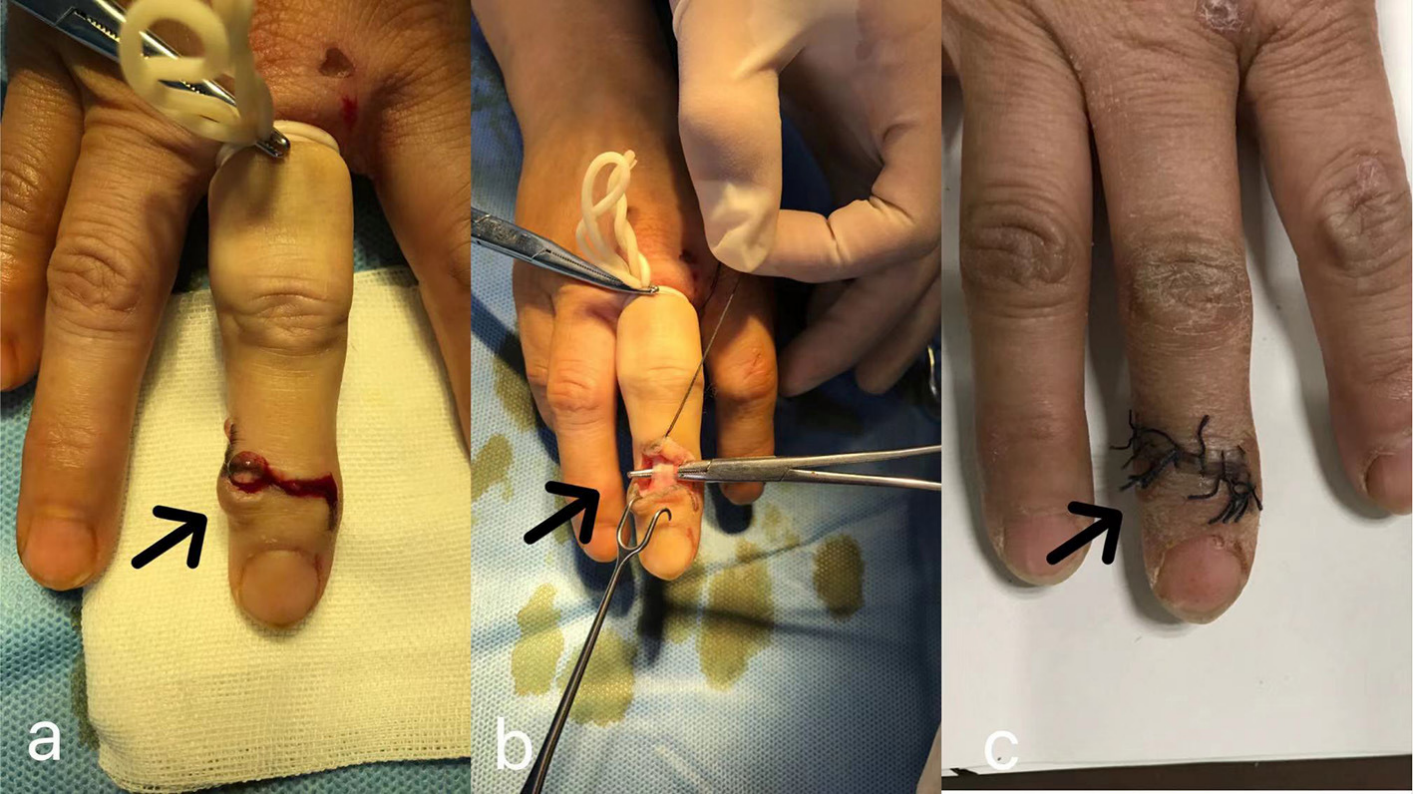
Resection of DMC; **(a)** we use an “S” incision to thoroughly expose the joint capsule; **(b,c)** through the “S” incision in the middle of the cyst, remove the joint capsule and osteophyte on the dorsal side of the joint, protect the extensor tendon, and suture the incision after the operation.

The operation is performed under digital nerve block anesthesia. According to the actual location, size, and shape of the cyst, we try to avoid the location of the cyst and make an “S” incision on the dorsal side of the DIP joint. Then, we cut open the subcutaneous tissue of the skin and expose the dorsal joint capsule and extensor tendon of the DIP joint. At this time, you can usually see the cyst pedicle or partially concealed cyst. If you do not see the cyst pedicle, you do not have to look for it. After determining the collateral ligament and paying attention to protection, we removed all the dorsal joint capsules and fibrous tissue. At this time, the pedicle of the cyst was removed, the fluid flowed out of the cyst, and the cyst atrophied. The osteophyte of the DIP joint hyperplasia was removed completely and then smoothed out. If part of the osteophyte is found at or near the stop of the extensor tendon, you should carefully pull the extensor tendon to expose the osteophyte or make a small incision in the middle of the extensor tendon to expose the osteophyte, and gently remove the osteophytes. Special attention should be paid to protect the integrity of the extensor tendon and the stop, and palliative resection can be done if it cannot be completely removed. To avoid the residual tissue, such as bone fragments and joint capsules, we rinsed repeatedly with normal saline. After stopping the bleeding, suture the incision. If the extensor tendon injury is suspected or the osteophyte is removed around the stop point of the extensor tendon, external fixation of the DIP joint is given for 4 weeks (the same as hammer finger fixation). Functional exercise was performed under the guidance of the doctor 3 days after the operation, and the stitches were removed after the incision healed completely. The surgical procedure is shown in Figure 1.

### The Assessment of Clinical Outcomes

We collected the medical history and physical examination data of the patients included in the study. All patients were followed-up 1 week, 1 month, and 3 months after operation and then followed up every 3–6 months. The range of motion (ROM) and visual analog scale (VAS) score of DIP were evaluated 3 months after the surgery.

### Radiographic Evaluations

As shown in **Table 2**, the radiological examinations were performed pre-operatively and 3 months post-operatively.

### Statistical Analysis

Clinical outcomes were statistically analyzed using SPSS26 software (IBM, Armonk, NY, USA). Normally distributed measures were expressed as mean ± SD. One-way ANOVA and two-way repeated-measures ANOVA were used to compare between and within-group differences. Count data were expressed as percentages. The *p* < 0.05 is statistically significant.

## Results

### Patient Demographics

As shown in Table 1, a total of 19 sites of the fingers of these 15 patients (6 men and 9 women) were enrolled in the study from February 2017 to August 2020. The average follow-up time was 36.93 ± 10.7 months. All patients were diagnosed as DMC, such as seven cases of the thumb, eight cases of the middle finger, two cases of the index finger, and two cases of the ring finger.

**Table 1:** Patient demographics and perioperative parameters.

**Characteristic**	**No. of patients**
Male (*n*, %)	6 (40)
Female (*n*, %)	9 (60)
Affected finger (*n*, %)
Thumb	7 (36.8)
Middle finger	8 (42.1)
Index finger	2 (10.5)
Ring finger	2 (10.5)
Follow-up (months)	36.93 ±10.7

### Outcomes of Clinical Data

The patient-reported outcomes are shown in Table 2. Of the 19 incisions on the fingers of these 15 patients, 18 incisions met the criteria for primary healing and 1 had skin necrosis around the incision. It healed smoothly after the dressing change. The patients were followed up for 12–54 months. The average follow-up time was 36.93 ± 10.7 months. There was no rupture of the extensor tendon and cyst recurrence, ROM of the affected finger was increased (ROM: 67.60 ± 5.40 vs. 71.27 ± 7.06, *p* > 0.05), and the pain of finger activity was improved (VAS score: 4.93 ± 0.88 vs. 4.07 ± 1.03, *p* < 0.05).

**Table 2:** Functional score.

	**Results**
VAS
Pre-operative	4.93 ± 0.88
3 months post-operatively	4.07 ± 1.03
ROM
Pre-operative	67.60 ± 5.40
3 months post-operatively	71.27 ± 7.06

*VAS, visual analog scale, ROM, range of motion*.

### Radiological Data

**Figure 2:**
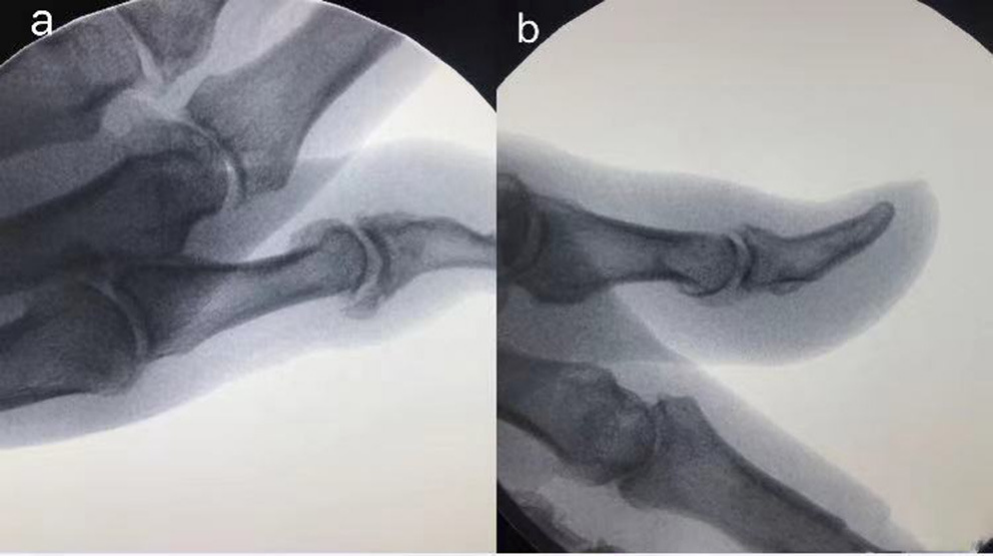
The imaging examination of the patient before and after surgery. **(a)** Pre-operative X-ray showed a large number of osteophytes in the DIP joint of the thumb; **(b)** X-ray showed osteophyte disappeared 3 months after the operation.

Imaging examination was performed before the surgery and 3 months post-surgery (Figure 2). X-ray showed that all the affected fingers were related to arthritis and there were osteophytes in different degrees on the dorsal side. After surgery, the osteophyte on the dorsal side of the affected finger disappeared.

## Discussion

It is generally believed that DMC is caused by degenerative changes in the fibrous capsule of synovial tissue in DIP. The incidence of mucoceles in OA ranges from 64 to 93% (16). OA and osteophytes play a role in promoting DMC (5). DMC may be caused by an outpouching of the synovial lining of a joint, or by mucus degeneration of connective tissue around the joint and accumulation of by-products of collagen proteolysis in the cyst (2). Therefore, the treatment of osteophyte and degenerative fibrous capsules is particularly important in the surgical treatment of DMC.

Common surgical procedures include cyst resection, bone spur resection, cyst drainage and bone spur resection, and skin flap repair after cyst resection (15.17). There is no simultaneous treatment for the etiology of osteophyte and degenerative fibrous capsules in arthritis. Due to the lack of attention of some patients, the cyst is often large and the surface of the skin is thin, which leads to skin necrosis and defects caused by simple resection of the cyst, which needs to be repaired by a skin graft or skin flap, which increases the difficulty and injury of the surgery. Simple resection of the cyst did not remove the degenerative fibrous tissue and joint osteophyte, resulting in a recurrence rate of over 10% (17). We learned through follow-up that our current method did not cause any patient recurrence.

We use an 'S' incision to thoroughly expose the joint capsule and remove all the fibrous tissue and osteophytes of the dorsal joint capsule, leaving only the extensor tendon and ligament. After resection, the contents of the cyst are drained into the joint without the need to remove the cyst. While the osteophyte is removed, the fibrous tissue that leads to the degeneration of DMC is removed completely, which will avoid the recurrence of DMC to the greatest extent. Richert et al. classified DMC as follows: the distal end of the cyst communicated with the interphalangeal joint, not with the interphalangeal joint and sub-matricial type. For type 1 and type 2, DMC originates from the fibrous tissue of the articular capsule, and complete removal of osteophyte and fibrous tissue of the articular capsule can avoid recurrence. For sub-matricial types, Gingrass et al. confirmed that the removal of osteophytes and internal drainage alone can achieve better results (8). When the dorsal fibrous capsule and other tissues were completely removed, the connection between the cyst and the articular capsule was completely opened, and the fluid in the cyst was drained, which immediately became smaller or even disappeared, and the tumor disappeared completely after an operation and was replaced by normal skin tissue (15). Some scholars believe the cyst communicates with the joint in all DMC (19).

When the skin of the cyst area of some patients is thin or even damaged, the incision should avoid the skin of the cyst area as far as possible; if it cannot be avoided, the skin should be cut under the skin together, and the cyst should not be peeled off and post-operative skin necrosis can be avoided to the greatest extent. In one case, the incision passed through the edge of the cyst and a small amount of skin necrosis occurred at the cyst after an operation, which was caused by the larger cyst and thinning of the surface skin.

Some of the affected fingers have mallet finger deformity before the operation, and the osteophyte at the stop of the extensor tendon will lift the extensor tendon, resulting in the relaxation of the extensor tendon. Therefore, the injury of collateral ligament and extensor tendon should be avoided during the operation, especially when the osteophyte is removed at the end of the extensor tendon, the removal of the osteophyte may lead to the injury of the extensor tendon, and lead to the occurrence of hammer finger. In this group, there was 1 case of hammered finger after the operation. The possible reasons are as follows: (1) the osteophyte near the stop of the extensor tendon will lift the extensor tendon, and the removal of the osteophyte will lead to the relaxation of the extensor tendon; (2) during the operation, the extensor tendon was overstretched to remove the osteophyte, resulting in tendon relaxation. In future cases, we should pay attention to protecting the stop of the extensor tendon to avoid excessive removal of osteophytes and avoid excessive traction. After surgery, the DIP joint was fixed in a straight or skip extension position for 4 weeks to protect the extensor tendon and avoid hammering finger caused by tendon relaxation or stop injury. There was no post-operative hammering finger in subsequent cases. Some scholars use Kirschner wire to fix DIP 4 weeks after osteophyte removal to protect the extensor tendon (15) or DIP fixation, such as a brace to protect extensor tendon (18).

The simultaneous appearance of OA of DMC and DIP, accompanied by pain, deformity, and decreased range of motion of the joint, are all factors that prompt the patient to seek medical treatment. The pain, deformity, and decreased mobility of DIP are caused by arthritis, so surgery for cysts alone will not relieve the pain, which is clearly explained to the patients before operation. For advanced DIP arthritis, arthrodesis can be used to treat arthritis pain. Joint fusion not only affects the movement of the joint but also has many related complications, such as bone non-union and infection. For DIP arthritis, the removal of osteophyte and degenerated joint capsule tissue can theoretically reduce intra-articular pressure, reduce the increase of synovial fluid caused by osteophyte stimulation, and avoid osteophyte obstruction during movement of joint. From the VAS score of patients before and after surgery, this can reduce DIP pain, which is consistent with previous studies by Thornburg et al. (2). Unfortunately, in one case, the patient suffered a serious decrease in ROM of the joint after the operation due to the injury of the extensor tendon. As a result, the ROM score of the patients before and after the operation was not statistically significant. However, in other patients, our method has improved the ROM of patients to some extent. Therefore, we believe that our method can increase the ROM of the joint and prolong the service life of the joint.

The joint deformities associated with DMC are usually mild hammering fingers caused by extensor tendon lengthening and the joint is tilted to one side. The osteophyte resection and joint debridement we used did not improve the degree of joint deviation and may prolong the length of the extensor tendon to some extent. Although most of the extensor tendons can recover to the pre-operative level. How to improve the deviation of the joint and shorten the extensor tendon to increase the ROM of the joint during the removal of DMC is another challenge for this kind of surgery. In addition, due to the existence of OA, even with osteotomy and joint debridement, long-term osteophyte and fibrous capsule tissue may reappear, which may lead to a recurrence of DMC. Due to the short follow-up time, the possibility of recurrence cannot be ruled out in this group of cases (16).

## Conclusion

In the treatment of DMC with osteophyte excision and joint debridement, the cyst is not removed but internal drainage is used, which reduces the complications, such as skin necrosis and large wound after excision. We removed the osteophyte and degenerative joint capsule tissue in DIP, which not only avoided the recurrence of DMC to the greatest extent but also reduced the joint stimulation caused by osteophyte and degenerative tissue. Although there is a patient with reduced ROM due to extensor tendon injury, our method generally reduces the pain of DIP and increases the ROM, which is a better surgical method for the treatment of DMC.

## Data Availability Statement

The raw data supporting the conclusions of this article will be made available by the authors, without undue reservation.

## Ethics Statement

Written informed consent was obtained from the individual(s) for the publication of any potentially identifiable images or data included in this article.

## Author Contributions

ZF and ZZ made substantial contributions to the design of this study and revised the manuscript. XS and LC collected the data. ZF, BY, and ZZ analyzed the data. ZF wrote the manuscript. All authors read and approved the final manuscript. All authors contributed to the article and approved the submitted version.

## Conflict of Interest

The authors declare that the research was conducted in the absence of any commercial or financial relationships that could be construed as a potential conflict of interest.

## Publisher's Note

All claims expressed in this article are solely those of the authors and do not necessarily represent those of their affiliated organizations, or those of the publisher, the editors and the reviewers. Any product that may be evaluated in this article, or claim that may be made by its manufacturer, is not guaranteed or endorsed by the publisher.

## Abbreviations

DMC: Mucous cyst of the distal interphalangeal joint
DIP: distal interphalangeal
VAS: visual analog scale
OA: osteoarthritis
ROM: range of motion.
